# Cytomegaloviruses in a Community of Wild Nonhuman Primates in Taï National Park, Côte D’Ivoire

**DOI:** 10.3390/v10010011

**Published:** 2017-12-29

**Authors:** Augustin Etile Anoh, Sripriya Murthy, Chantal Akoua-Koffi, Emmanuel Couacy-Hymann, Fabian Hubertus Leendertz, Sébastien Calvignac-Spencer, Bernhard Ehlers

**Affiliations:** 1 UFR de Biosciences/Laboratoire de Zoologie et Biologie Animale, Université Felix Houphouët Boigny, Abidjan BP 1174, Cote D’Ivoire; anohethyl@yahoo.fr; 2 Centre de Recherche pour le Développement, Université Alassane Ouattara, Bouaké BP 1174, Cote D’Ivoire; Akouamc@yahoo.fr; 3 Division 12 Measles, Mumps, Rubella and Viruses Affecting Immunocompromised Patients, Robert Koch Institute, Seestrasse 10, 13353 Berlin, Germany; murthysripriya89@gmail.com; 4 Laboratoire National D’appui au Développement Agricole/Laboratoire Central de Pathologie Animale, Bingerville BP 206, Cote D’Ivoire; chymann@hotmail.com; 5 Epidemiology of Highly Pathogenic Microorganisms, Robert Koch Institute, 13353 Berlin, Germany; leendertzf@rki.de; 6 Viral Evolution, Robert Koch Institute, 13353 Berlin, Germany

**Keywords:** cytomegalovirus, nonhuman primate, genetic diversity, host specificity, co-divergence, Taï National Park, Côte d’Ivoire

## Abstract

Cytomegaloviruses (CMVs) are known to infect many mammals, including a number of nonhuman primates (NHPs). However, most data available arose from studies led on captive individuals and little is known about CMV diversity in wild NHPs. Here, we analyzed a community of wild nonhuman primates (seven species) in Taï National Park (TNP), Côte d’Ivoire, with two PCR systems targeting betaherpesviruses. CMV DNA was detected in 17/87 primates (4/7 species). Six novel CMVs were identified in sooty mangabeys, Campbell’s monkeys and Diana monkeys, respectively. In 3/17 positive individuals (from three NHP species), different CMVs were co-detected. A major part of the glycoprotein B coding sequences of the novel viruses was amplified and sequenced, and phylogenetic analyses were performed that included three previously discovered CMVs of western red colobus from TNP and published CMVs from other NHP species and geographic locations. We find that, despite this locally intensified sampling, NHP CMVs from TNP are completely host-specific, pinpointing the absence or rarity of cross-species transmission. We also show that on longer timescales the evolution of CMVs is characterized by frequent co-divergence with their hosts, although other processes, including lineage duplication and host switching, also have to be invoked to fully explain their evolutionary relationships.

## 1. Introduction

Cytomegaloviruses (CMVs; family *Herpesviridae*; genus *Betaherpesvirinae*) are viruses with a double-stranded DNA genome of >200 kbp. In humans, CMV commonly causes asymptomatic infection in immunocompetent individuals, but the virus is a major cause of morbidity and mortality in newborns and immunosuppressed patients who are less able to counter primary infection or reactivation of latent virus [1,2,3,4,5]. Fever, hepatitis, pneumonia, dyspnea, gastrointestinal disorders (diarrhea), retinitis and neurological disorder are usually the clinical signs encountered [6,7]. CMVs also infect many nonhuman mammals, e.g., nonhuman primates (NHPs) and rodents. If and which diseases are caused by CMVs in NHPs is, to our knowledge, not known [8].

NHP CMVs have been detected by PCR and serology or isolated in cell culture mostly from captive individuals, i.e., hominines, including chimpanzees and orangutans [9,10] and Old World monkey species, e.g., rhesus macaques [11], cynomolgus macaques [12], Formosan rock macaques [13], African green monkeys [14], drills [15], and baboons [16]. In very few studies, CMVs have been identified in wild NHPs, i.e., chimpanzees, gorillas and two species of colobus monkeys [10,17,18]. In all cases, CMVs infecting NHPs appeared to be host-specific.

However, infection studies in cell culture suggest that the species barrier for NHP CMVs is not absolute. Rhesus CMV displays some capacity to replicate in cells of other NHPs and in cells of human origin [19,20]. Chimpanzee CMV has been grown on human fibroblasts, lung and brain cells [21,22] and human CMV on chimpanzee fibroblasts [23]. One possible explanation for this discrepancy is that we know very little about CMV genetic diversity and their distribution in wild NHPs. First insight came from a recent study carried out in Taï National Park (TNP), Côte d’Ivoire, in which a primate predator-prey system—western chimpanzees (*Pan troglodytes verus*), and their primary (western red colobus; *Piliocolobus badius*) and secondary prey (black-and-white colobus; *Colobus polykomos*)—was used to study the risk of herpesvirus transmission between different primate species in the wild [17]. Although the colobus monkeys were frequently infected with CMVs and different gammaherpesviruses, there was no evidence for cross-species herpesvirus transmission, despite frequent exposure of the chimpanzees to their herpesvirus-infected monkey prey. It was suggested that interspecies transmission of beta- and gammaherpesviruses between members of different NHP species is at most a rare event in the wild. In a subsequent report, sequences of human CMVs, but no NHP CMVs, were detected in oral swabs and stool samples of human individuals living at the border of TNP [24]. This also suggests that zoonotic events are at most rare since people in the region still hunt and consume NHPs and are therefore also frequently exposed to infected NHP tissues [25]. Finally, fecal samples from central chimpanzees (*Pan troglodytes troglodytes*) and western lowland gorillas (*Gorilla gorilla gorilla*) from Odzala-Kokoua National Park (OKNP), Republic of Congo, were shown to be positive for CMVs of chimpanzee and gorilla, respectively, but negative for CMVs of other species [18].

Here we extended these tests for cross-species transmission of CMVs by targeting an entire wild primate community comprised of seven primate species living syntopically in TNP. Altogether, CMVs have been detected in six out of seven primate species including multiple CMV infections in single individuals, but no infection of multiple host species with the same CMV.

## 2. Materials and Methods

### 2.1. Field sites and Sample Collection

Necropsy and blood samples were collected over a period of more than 10 years from deceased or live individuals of 8 NHP species (great apes and Old World monkeys); all necropsies were performed on carcasses detected opportunistically, i.e., no primate was culled for this project. Sampling was done in TNP in Cote d’Ivoire, the largest protected block of tropical moist forest in West Africa. Because of the history of anthrax and Ebola in NHP populations of TNP, full body protection suits and masks were required and mandatory for sampling. The field samples were initially snap frozen in liquid nitrogen and then stored at −80 °C at LANADA (Bingerville, Côte d’Ivoire), before being transported on dry ice to Robert Koch Institute, Germany. Collection of NHP samples was conducted with permission of the Ministry of Research of Côte d’Ivoire and the Office Ivoirien des Parcs et Réserves (OIPR).

### 2.2. DNA Extraction

Total nucleic acid was extracted from necropsy samples (organs) and whole blood or buffy coat using the DNAeasy blood and tissue Kit (Qiagen, Hilden, Germany), according to the manufacturer’s instructions. DNA concentration was quantified using Nanodrop ND-1000 Spectrophotometer (Nanodrop Technologies, Santa Clara, CA, USA) or Qubit (ThermoFischer, Waltham, MA, USA).

### 2.3. Generic PCR

**Figure 1:**
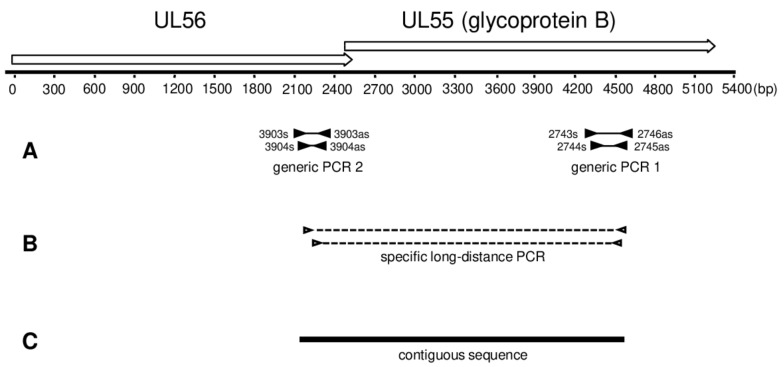
Map of targeted open reading frames and diagram of PCR strategy. Degenerate nested primers (black triangles) were used to amplify part of the UL55 or UL56 open reading frame. The amplified fragments are represented by thin solid lines between the primer binding sites (**A**); Long-distance nested PCR was performed with specific primers (open triangles). The amplified fragments are represented by dashed lines between the primer binding sites (**B**); Fragments amplified in (**A**,**B**) were sequenced and assembled to a final contiguous sequence of 2.2 to 2.4 kb (**C**). At the top of the figure, the genomic locus spanning open reading frames UL55 and UL56 is depicted with open arrows. The arrowhead indicates the direction of transcription. The start of the ruler corresponds to the first base of the ORF UL56.

For amplification of conserved NHP CMV UL55 and UL56 sequences, two independent generic nested PCRs were performed targeting either UL55 (glycoprotein B) (PCR 1; Figure 1A) or UL56 (PCR 2; Figure 1A) coding sequences using degenerate primer sets (Table S1). These primers had been designed in order to detect CMV-like betaherpesviruses, including those of NHP and humans. Using these primers, several primate and rodent CMV-like viruses have been discovered [10,17,26]. The PCRs were performed here as published previously [17,25].

### 2.4. Long-Distance Nested PCR

Long distance nested PCR was carried out using the TaKaRa-EX PCR kit (Takara Bio Inc., Otsu, Japan) according to the manufacturer’s instructions, with an annealing temperature of 50 °C. Sequences of primers used are listed in Table S2. PCR mix was set up on ice and contained as template 250 ng of DNA sample that tested positive for CMV with both PCR 1 and 2. Reaction product had a length of 2.2–2.3 kb (Figure 1B).

### 2.5. Cytochrome *B* PCR

The host species assignment of CMV-positive samples was confirmed by the PCR amplification of a fragment of the mitochondrial cytochrome *b* (*cytb*) gene, as done previously [26].

### 2.6. Sequencing

All PCR products were sequenced on both strands according to Sanger’s methodology. For each novel virus, the sequences of PCR 1, PCR 2, and long-distance PCR were assembled to a final contiguous sequence of 2.2–2.4 kb (Figure 1).

### 2.7. BLAST Analysis

Blast search was undertaken in the NCBI database (www.ncbi.nlm.nih.gov/BLAST) [27].

### 2.8. Phylogenetic and Co-Phylogenetic Analysis

The translated amino acid UL55 sequences generated in the course of this study were aligned with other publicly available UL55 sequences derived from NHPs using Muscle [28] as implemented in Seaview v4 [29]. Conserved amino acid blocks were identified using gblocks (also implemented in Seaview) [30]. The final alignment included 36 CMV sequences and one outgroup sequence (human herpesvirus 6B; KT246127); it was comprised of 543 positions. We then performed model selection using Prottest v2.4 [31], focusing on amino acid models available in BEAST (see below) and employing a full maximum likelihood (ML) optimization procedure. Using the Bayesian information criterion we identified LG + G as the model to which our data were best-fit. We then ran phylogenetic analyses using both ML and Bayesian Markov chain Monte Carlo (BMCMC) approaches. For the ML analysis, we used PhyML v3 [32], as implemented on the PhyML webserver [33]; tree search was performed using the BEST algorithm and branch support was estimated using Shimodaira-Hasegawa-like approximate likelihood ratio tests (SH-like aLRT; [34]). BMCMC analyses were run using BEAST v1.8.2 assuming a relaxed lognormal molecular clock and modeling tree shape according to a birth-death speciation model [35]. The output of multiple runs was examined for convergence and appropriate sampling of the posterior with Tracer v1.6 (http://tree.bio.ed.ac.uk/software/tracer/) before being merged using LogCombiner v1.8.2 (distributed with BEAST). The best representative tree was identified from the posterior set of trees and annotated with TreeAnnotator v1.8.2 (also distributed with BEAST).

We downloaded the host tree from the 10kTrees webserver [36]. The 10kTrees webserver provides pre-computed BMCMC timetrees for several taxa, including Primates.

We ran co-phylogenetic analyses using Jane v4 [37]. Jane implements a genetic algorithm to quickly identify the most parsimonious scenarios of co-evolution, involving several types of events (co-divergence, duplication, duplication with host switch, loss and failure to diverge). Host and parasite phylogenies have to be provided along with the according tip mapping and an event cost matrix. A simplified version of the CMV phylogeny was used as input, whereby single-host clades were collapsed; to be conservative regarding the number of co-speciation events, we used the ML tree, in which a couple of host-specific association identified by the BMCMC analysis were not supported. The cost set we used defined co-divergence events as having a cost −1 and all non co-divergence events as having no cost. This allows equating costs and co-divergence events. Jane was run using the vertex-based cost mode and the parameters of the genetic algorithm were kept at their default values (population size 100, number of generations 100). To determine the probability of observing the inferred costs by chance, costs were also calculated on a set of 500 samples for which tip mapping was randomized. Settings of the genetic algorithm were kept at default values.

## 3. Results

### 3.1. Detection of Cytomegaloviruses in Wild Nonhuman Primates from TNP

We tested DNA extracts from 87 NHP individuals (representing 7 NHP species; confirmed by cytochrome *b* sequencing) for the presence of CMVs using a nested PCR sytem generic for members of the herpesvirus subfamily *Betaherpesvirinae* (nested PCR 1; Table S1 and Figure 1A). The sequences derived from the amplicons obtained were analyzed by BLASTN and BLASTX searches. The detection of CMV DNA was confirmed in 17/87 NHPs: 7/37 *Cercocebus atys* (sooty mangabey), 1/4 *Cercopithecus campbelli* (Campbell’s monkey), 1/8 *Cercopithecus diana* (Diana monkey) and 9/18 *Pan troglodytes verus* (western chimpanzee) (Table 1).

Names and abbreviations for newly detected viruses were formed from host species and genus names to which the pathogen was assigned, the term “cytomegalovirus” and a consecutive number, e.g., Cercocebus atys cytomegalovirus 1 (CatyCMV1) (Table 2).

None of the *Colobus polykomos*, *Perodicticus potto* (potto) and *Piliocolobus badius*, individuals tested in this study were PCR-positive. However, positive black-and-white and red colobus individuals had been identified in a previous study led in TNP [17], which means we have now identified CMVs in 6/7 NHP species living in this ecosystem (Table 2).

Except whole blood samples (PCR-negative), 10–28% of the organ and body fluid samples (kidney, liver, lung, spleen, heart, gastrointestinal tract, lymph node, buffy coat) were CMV-positive in nested PCR 1 (Table 3). In 3/17 CMV-positive individuals (from three NHP species), different viruses were detected in different tissues. Furthermore, PtroCMV1 and PtroCMV2 were co-detected in both spleen and liver of *Pan troglodytes verus*; CatyCMV1 and CatyCMV2 in both lung and liver of *Cercocebus atys*; CatyCMV1 and CatyROV1 in kidney of *Cercocebus atys*; CcamCMV1 and CcamCMV2 in kidney and duodenum of *Cercopithecus campbelli*. None of the viruses was detected in members of multiple species (Table 4).

We applied a two-step approach to extend the short partial UL55 (glycoprotein B encoding) sequences that had been amplified with nested PCR 1, into the adjacent coding sequence (UL56). First, nested PCR 2 (which amplifies a short part of UL56 coding sequence; Table S1 and Figure 1a) was applied to all CMV-positive DNA extracts. This was successful for 20/23 DNA extracts (data not shown). Second, on the basis of the partial UL55 and UL56 sequences nested primers were specifically selected for each novel virus (Table S2) and used for overlapping long-distance nested PCR (LD-PCR) (Figure 1B), spanning 2.2–2.3 kb. Out of 20 extracts, 14 gave rise to the respective LD-PCR product. These products were purified and sequenced, and the LD-PCR sequences assembled with the short UL55 and UL56 sequences, to give final contigs of 2.23–2.39 kb that include 1.7–1.8 kb of the UL55 coding sequence (Figure 1C). These comprised all CMV-positive NHP species, and with the exception of CatyCMV3, all CMVs detected in this study (Table 2).

The novel CMV sequences were submitted to GenBank and are available under the accession numbers listed in Table 2.

### 3.2. Phylogeny and Co-Phylogeny of Cytomegaloviruses and Their Hosts

**Figure 2:**
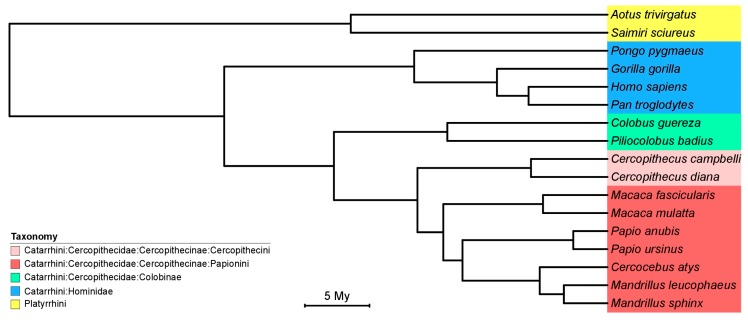
Time tree of nonhuman primate host species. This time tree was produced using a precompiled set of posterior trees available at the 10KTrees webserver [36]. Host common names are as follow: *Aotus trivirgatus* (three-striped night monkey), *Cercocebus atys* (sooty mangabey), *Cercopithecus campbelli* (Campbell’s monkey), *Cercopithecus diana* (Diana monkey), *Colobus guereza* (guereza), *Colobus polykomos* (black-and-white colobus), *Gorilla gorilla* (Western lowland gorilla), *Macaca fascicularis* (cynomolgus monkey), *Macaca mulatta* (rhesus macaque), *Mandrillus sphinx* (mandrill), *Mandrillus leucophaeus* (drill), *Pan troglodytes* (chimpanzee)*, Papio anubis* (olive baboon), *Papio ursinus* (chacma baboon), *Piliolobus badius* (western red colobus), *Pongo pygmaeus* (Borneo orangutan), *Saimiri sciureus* (common squirrel monkey).

**Figure 3:**
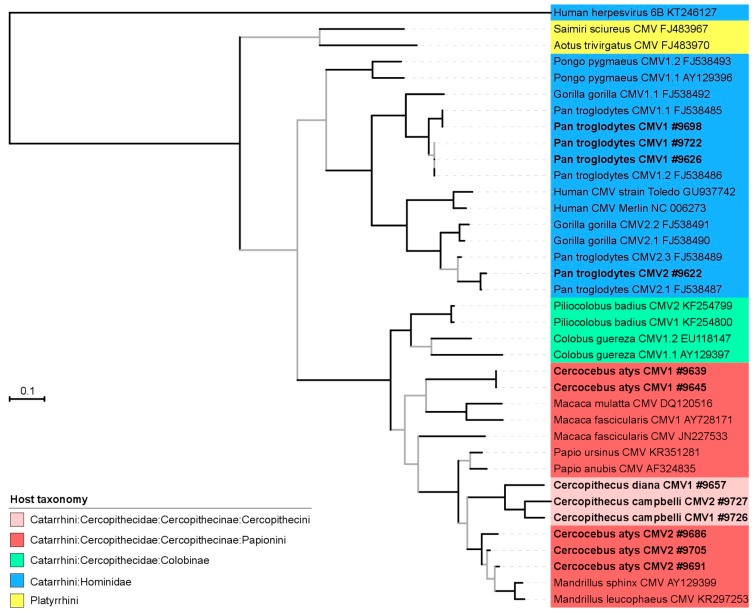
Maximum likelihood tree of partial CMV UL55 amino acid sequences. Sequences generated for this study are bold and displayed with their sample number (accession numbers in Table 1). Internal branches supported by Shimodaira-Hasegawa-like approximate likelihood ratio test (SH-like aLRT) values <0.95 are grey. The common names of the hosts after which the viruses are named are given in the legend of Figure 1.

**Figure 4:**
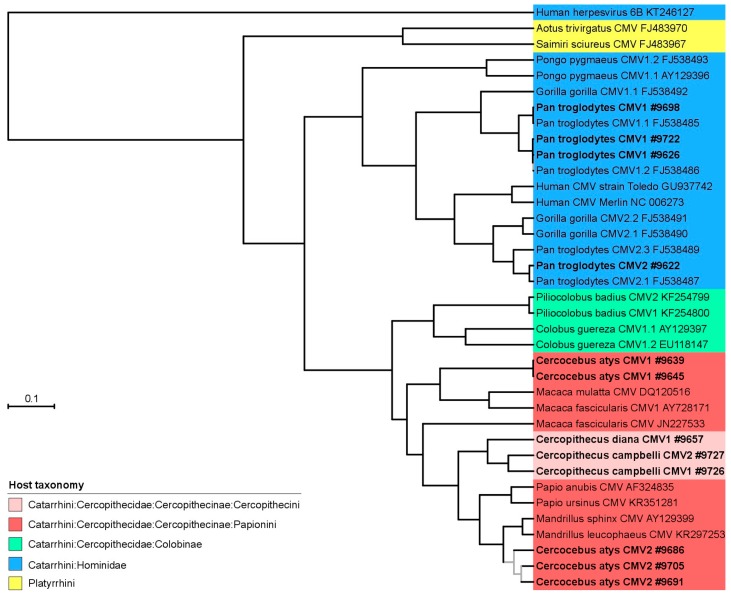
Bayesian Markov chain Monte Carlo tree of partial CMV UL55 amino acid sequences. Sequences generated for this study are bold and displayed with their sample number (accession numbers in Table 1). Internal branches supported by posterior probabilities <0.95 are grey. The common names of the hosts after which the viruses are named are given in the legend of Figure 1.

We used the new CMV sequences to investigate the diversification of CMV and more particularly how it was influenced by their host diversification. To compare the host and CMV diversification we generated phylogenetic trees from pre-computed resources (host phylogeny; Figure 2) or an alignment of CMV UL55 sequences comprising our new sequences and other publicly available sequences derived from nonhuman primates which we analyzed in a maximum likelihood or Bayesian framework (Figure 3 and Figure 4, respectively).

CMV phylogenetic relationships reflected their host diversification in many instances, and at multiple taxonomic levels (Figure 2, Figure 3 and Figure 4). Thus, CMVs infecting platyrrhines (New World monkeys) and catarrhines (apes and Old World monkeys) formed monophyletic groups, as did CMVs infecting members of the two catarrhine families represented in the trees (Cercopithecidae and Hominidae). Within these families, CMVs infecting members from the subfamilies Cercopithecinae and Colobinae were monophyletic, as were those infecting orangutans and African great apes. At an even lower taxonomic level, CMVs infecting monkeys belonging to the tribe Cercopithecini also formed a clade. Finally, when multiple CMVs were detected in a same host species, they generally formed monophyletic groups, i.e., CMVs found in *Cercopithecus campbelli*, *Colobus guereza*, *Piliocolobus badius* or *Pongo pygmaeus*. All in all, the comparison of the phylogenetic trees of CMV and their hosts therefore supported the notion that CMV diversification was strongly influenced by the diversity or diversification of their NHP hosts.

However, a number of exceptions were also observed in both ML and BMCMC CMV trees. As already pinpointed elsewhere, chimpanzees and gorillas appeared to be infected with CMVs belonging to a minimum of two lineages, suggesting either a viral lineage duplication event in the ancestor of African great apes or a more complex scenario involving multiple host switches between African great ape species [10]. Our phylogenetic analyses also showed that CMVs infecting *Cercocebus atys* belong to a minimum of two lineages, very distantly related to one another. In addition, CMVs infecting members of the Cercopithecini tribe, while exhibiting a phylogenetic placement that differed in the ML and BMCMC analyses, always disrupted the monophyly of CMVs infecting members of the Papionini tribe. This suggests again that pure co-divergence cannot fully explain the distribution of CMV genetic diversity in their hosts. Alternative processes, including lineage duplication, lineage loss and host switch are required to resolve discrepancies with the host phylogeny.

To further quantify the relative weight of co-speciation and these other processes during the diversification of NHP CMVs, we also ran explicit co-phylogenetic analyses. These analyses identified 8838 most parsimonious scenarios that all came at the same cost (−16) and all involved 16 co-speciation events and 10 non co-speciation ones (tip mapping randomization test; *p* = 0).

## 4. Discussion

Our systematic search for CMVs in the NHP community of TNP investigated seven NHP species. Eight CMVs were detected in four species, six of them novel and representing the first CMV detection in *Cercocebus atys*, *Cercopithecus campbelli* and *Cercopithecus diana*. Combined with the previous study of Murthy et al. on three NHP species in TNP [17], ten distinct CMVs were identified in six NHP species (Table 1). The only species for which no CMV could be identified was the potto but it was also the species for which sampling intensity was the lowest (*n* = 2). Collectively, these results therefore indicate that most NHP species in TNP are enzootically infected with CMVs.

In line with the moderate to high prevalence observed, we also found that three of the 17 CMV-positive NHP individuals (1 chimpanzee, 1 sooty mangabey, 1 Campbell’s monkey) were infected with at least two different CMVs. This adds to accumulating evidence in the literature highlighting the commonness of superinfection with different CMVs in healthy people [38,39,40], chimpanzees [10,17], and rhesus macaques [41,42,43].

None of the eight CMVs detected in TNP infected more than one NHP species. This was also observed in previous studies led on wild NHPs: CMVs were only detected in their respective host species in chimpanzees, gorillas and two species of colobus monkeys [10,17,18]. However, our results considerably reinforce the notion that cross-species CMV transmission is at most rare in extending this observation to an entire primate community and to different modalities of NHP interactions. For example, a number of monkeys considered in this study form polyspecific groups, which could increase the likelihood of cross-species CMV transmission.

Our data also allowed us to investigate how the diversification patterns of CMVs and their hosts are correlated. Even considering the phylogenetic tree which was the least favorable to co-speciation events (the ML one) we found that co-divergence events dominate over non co-divergence events (61% vs. 39%). Although we did not specifically investigate it, the comparison of relative divergence dates in the CMV and NHP trees seems suggestive of a similar diversification pace (even though not completely overlapping). It therefore seems likely that these apparent co-divergence events might indeed represent *bona fide* divergence events, pinpointing a very long-term association of these viruses and their hosts and measurable host tracking by CMVs. Interestingly, this mixture of diversification processes dominated by co-divergence seems to be relevant to a number of viruses with a double-stranded DNA genome, e.g., [44,45,46].

The combination of: (i) generally benign CMV infections in immunocompetent hosts; (ii) possibility to induce strain-specific immunity by superinfecting seropositive individuals with new CMVs; and (iii) apparent strong host-specificity has prompted efforts aimed at evaluating CMVs as platforms for self-disseminating vaccines. Recently, CMVs of NHPs have notably been considered as potential vaccine platform to protect NHPs in remote areas (and indirectly also humans) against cross-species transmission of lethal infectious agents such as ebolaviruses [47]. Although our ecological and evolutionary analyses of the distribution and diversification of CMVs in their natural hosts seem broadly compatible with the abovementioned prerequisites, they also pinpoint that non co-divergence events have left a measurable trace in the CMV phylogeny, which suggests that these approaches will at the very least require a very careful risk assessment.

## Figures and Tables

**Table 1:** Cytomegalovirus (CMV) prevalence in nonhuman primates in Taï National Park, Côte d’Ivoire.

Common Host Name	Host Species	Number of Individuals Tested	Number of Positives	% CMV Prevalence (95% CI ^§^)	References
Western chimpanzee	*Pan troglodytes verus*	38 (18 *)	15 (9 *)	0.39 (0.24–0.57)	[17]
Sooty mangabey	*Cercocebus atys*	37 *	7 *	0.19 (0.09–0.36)	
Western red colobus	*Piliocolobus badius*	31 (13 *)	4 (0 *)	0.13 (0.04–0.31)	[17]
Black-and-white colobus	*Colobus polykomos*	12 (3 *)	1 (0 *)	0.08 (0.01–0.40)	[17]
Diana monkey	*Cercopithecus diana*	8 *	1 *	0.12 (0.01–0.53)	
Campbell’s monkey	*Cercopithecus campbelli*	4 *	1 *	0.25 (0.01–0.78)	
Potto	*Perodicticus potto*	2 *	0 *	0 (0–0.90)	
Total		132	29	22	

* Individuals tested in the current study; ^§^ Confidence interval.

**Table 2:** Cytomegaloviruses of nonhuman primates in Taï National Park, Côte d’Ivoire.

Host Family and Species	Sample Number	Full Virus Name	Abbreviated Virus Name	UL55 Sequence (kb)	References	Accession Number
Hominidae						
*Pan troglodytes verus*	9595	Pan troglodytes cytomegalovirus 1	PtroCMV1	2.34	Current study	MG593784
	9626			2.34		MG593786
	9698			2.36		MG593793
	9722			2.34		MG593797
	3147			2.79	[10]	FJ538485
	9622	Pan troglodytes cytomegalovirus 2	PtroCMV2	2.39	Current study	MG593785
	2296			1.83	[10]	FJ538487
Cercopithecidae						
*Cercocebus atys*	9639	Cercocebus atys cytomegalovirus 1	CatyCMV1	2.23	Current study	MG593787
	9645			2.23		MG593788
	9646			2.23		MG593789
	9686	Cercocebus atys cytomegalovirus 2	CatyCMV2	2.24	Current study	MG593791
	9691			2.25		MG593792
	9705			2.23		MG593794
	9706	Cercocebus atys cytomegalovirus 3	CatyCMV3	0.20	Current study	MG593795
	9720	Cercocebus atys roseolovirus 1	CatyROV1	0.22	Current study	MG593796
*Cercopithecus campbelli*	9726	Cercopithecus campbelli cytomegalovirus 1	CcamCMV1	2.24	Current study	MG593798
	9727	Cercopithecus campbelli cytomegalovirus 2	CcamCMV2	2.24	Current study	MG593799
*Cercopithecus diana*	9657	Cercopithecus diana cytomegalovirus 1	CdiaCMV1	2.24	Current study	MG593790
*Piliocolobus badius*	6944	Piliocolobus badius cytomegalovirus 1	PbadCMV1	1.67	[17]	KF254800
	6940	Piliocolobus badius cytomegalovirus 2	PbadCMV2	1.67		KF254799
	4598	Piliocolobus badius cytomegalovirus 3	PbadCMV3	0.22		KF318790

**Table 3:** Cytomegalovirus test results for organ and blood samples from nonhuman primates in Taï National Park, Côte d’Ivoire.

Tissue	*Cercopithecus diana*	*Cercopithecus campbelli*	*Cercocebus atys*	*Colobus polykomos*	*Pan troglodytes*	*Piliocolobus badius*	*Perodicticus poto*
Kidney	2	2 (1)	5 (1)	-	1	2	-
Liver	2	1	10 (2)	1	9 (3)	4	1
Lung	4 (1) ^a^	-	11 (3)	-	7 (1)	3	1
Spleen	1	1	5 (1)	1	11 (4)	1	2
Whole blood	2	-	9	2	-	-	-
Buffy coat	- ^b^	-	2 (1)	-	1	7	-
Heart	-	1	3 (1)	-	1	-	-
Gastrointestinal tract	2	1 (1)	3	-	1 (1)	-	-
Lymph node	-	2	2(1)	-	4 (1)	1	-
Total	13 (1)	3 (0)	50 (10)	4 (0)	37 (10)	18 (0)	4 (0)

^a^ number of samples tested; in parentheses: number of samples CMV-positive in betaherpesvirus-generic nested PCR; ^b^ hyphen: not available.

**Table 4:** Cytomegaloviruses in organs and blood of nonhuman primates in Taï National Park, Côte d’Ivoire.

Host Family and Species	Spleen	Blood/Buffy Coat	Lymph Node	Heart	Lung	Kidney	Liver	Oesophagus	Duodenum
Hominidae									
*Pan troglodytes verus*	PtroCMV1 ^a^ PtroCMV2	PtroCMV2	PtroCMV1		PtroCMV1		PtroCMV1 PtroCMV2	PtroCMV2	-
Cercopithecidae									
*Cercocebus atys*	CatyCMV1	CatyCMV2		CatyCMV3	CatyCMV1, CatyCMV2	CatyCMV1, CatyROV1	CatyCMV1, CatyCMV2		-
*Cercopithecus campbelli*	-	-	-	-	-	CcamCMV1	-	-	CcamCMV2
*Cercopithecus diana*					CdiaCMV1				
*Colobus polykomos*			-	-	-	-		-	-
*Perodicticus potto*		-	-	-		-		-	-
*Piliocolobus badius*				-		-		-	-

^a^ sequences of the indicated viruses were obtained from PCR products generated with degenerate nested primers that are generic for members of the Betaherpesvirinae (PCR 1); hyphen: PCR negative.

## Supplementary Materials

The following is available online at www.mdpi.com/1999-4915/10/1/11/s1, Table S1: Primers used for generic amplification of CMV UL55 and UL56 sequences; Table S2: Primers used for long-distance PCR amplification of CMV UL55/UL56 sequences.
