# Supplementary material for: Cytomegaloviruses in a Community of Wild Nonhuman Primates in Taï National Park, Côte D’Ivoire
Source: Viruses. 2017 Dec 29;10(1):11. doi: 10.3390/v10010011 (PMC5795424; doi:10.3390/v10010011)
Supplement: Supplementary file 1 [file viruses-10-00011-s001.pdf]

Table S1. Primers used for generic amplification of CMV UL55 and UL56 sequences.

PCR	Target Gene	Primer Name	PCR Round	Primer sequence 5′–3′	Product Length [c]	Binding Site in AD169 Genome
PCR1	UL55 (gB)	2743-s [a]	1st	CGCAAATCGCAGA(N/I [b])KC(N/I)TGGTG	320	81997–81976
		2746-as		TGGTTGCCCAACAG(N/I)ATYTCRTT		81679–81701
		2744-s	2nd	TTCAAGGAACTCAGYAARAT(N/I)AAYCC	230	81947–81922
		2745-as		CGTTGTCCTC(N/I)CC(N/I)ARYTG(N/I)CC		81698–81719
PCR 2	UL56	3903-s	1st	CCTGTCGCACAATGTGGACATG	250	84106–84085
		3903-as		CAGCTGTTTTCCGAA(N/I)GTTTCRTTAT		83857–83882
		3904-s	2nd	TGGCCTACGCYTGYGAYAACG	180	84086–84066
		3904-as		GCGAACGTGC(N/I)TCCACATCTCC		83908–83929

[a] s = sense; as = antisense; [b] I = Inosine; [c] approximate length.

Table S2. Primers used for long-distance PCR amplification of CMV UL55/UL56 sequences.

PCR	Primer	PCR Round	Primer Sequence 5′–3′	Target Virus
**LD PCR 1**	7399s	1st	ACCCTAGCGAGTGGATGGT	CatyCMV1
	7399as		CTCTGATCCACCTCCACRCACT	
	7400s	2nd	CGAGTGGATGGTGGTCAAGT	
	7400as		TGATCCACCTCCACRCACTTAGC	
**LD PCR 2**	7399s	1st	ACCCTAGCGAGTGGATGGT	CatyCMV2
	7401as		AGAATCTGTACAACACCACAGG	
	7402s	2nd	TGTGTGGATCTCAACATGTTGC	
	7402as		TCCTTCACATGCATGTCACG	
**LD PCR 3**	7403s	1st	GGTACACCCTAGCGAGTGGA	CcamCMV1, CcamCMV2, CdiaCMV1
	7403as		CCAGGTTCACCYTTYACAYGCA	
	7404s	2nd	ACTGTATTGATCTSAACATGTTGC	
	7404as		GGTTTGTCGTAGATGGCGGA	
**LD PCR 4**	7407s	1st	ACGATCTGGTCAAGTGCGC	PtroCMV1
	7407as		AGCACGTCGCCCATGAAG	
	7408s	2nd	CGAGTGGATGGTGGTCAAGT	
	7408as		ATGGGCTTGTCGTAGATGGC	
**LD PCR 5**	7409s	1st	GGTGTACCCCAGCGAGTG	PtroCMV2
	7409as		ACGCTGGTCTGGTTGATGTT	
	7410s	2nd	CGAGTGGATGGTGGTCAAGT	
	7410as		GTTGATGTTGACGCAGCTGG	
